# Financial Time Series Prediction Using Elman Recurrent Random Neural Networks

**DOI:** 10.1155/2016/4742515

**Published:** 2016-05-18

**Authors:** Jie Wang, Jun Wang, Wen Fang, Hongli Niu

**Affiliations:** ^1^School of Science, Beijing Jiaotong University, Beijing 100044, China; ^2^School of Economics and Management, Beijing Jiaotong University, Beijing 100044, China

## Abstract

In recent years, financial market dynamics forecasting has been a focus of economic research. To predict the price indices of stock markets, we developed an architecture which combined Elman recurrent neural networks with stochastic time effective function. By analyzing the proposed model with the linear regression, complexity invariant distance (CID), and multiscale CID (MCID) analysis methods and taking the model compared with different models such as the backpropagation neural network (BPNN), the stochastic time effective neural network (STNN), and the Elman recurrent neural network (ERNN), the empirical results show that the proposed neural network displays the best performance among these neural networks in financial time series forecasting. Further, the empirical research is performed in testing the predictive effects of SSE, TWSE, KOSPI, and Nikkei225 with the established model, and the corresponding statistical comparisons of the above market indices are also exhibited. The experimental results show that this approach gives good performance in predicting the values from the stock market indices.

## 1. Introduction

Predicting stock price index is difficult due to uncertainties involved. In the past decades, the stock market prediction has played a vital role for the investment brokers and the individual investors, and the researchers are on the constant look out for a reliable method for predicting stock market trends. In recent years, the artificial neural networks (ANNs) have been applied to many areas of statistics. One of these areas is time series forecasting. References [1–3] reveal different time series forecasting by ANNs methods. ANNs have been also employed independently or as an auxiliary tool to predict time series. ANNs are nonlinear methods which mimic nerve system. They have functions of self-organizing, data-driven, self-study, self-adaptive, and associated memory. ANNs can learn from patterns and capture hidden functional relationships in a given data even if the functional relationships are not known or difficult to identify. A number of researchers have utilized ANNs to predict financial time series including backpropagation neural networks, back radial basis function neural networks, generalized regression neural networks, wavelet neural networks, and dynamic artificial neural network [4–9]. Statistical theories and methods play an important role in financial time series analysis because both financial theory and its empirical time series contain an element of uncertainty. Some statistical properties for the stock market fluctuations are uncovered in the literatures such as power-law of logarithmic returns and volumes, heavy tails distribution of price changes, volatility clustering, and long-range memory of volatility [1, 10–12].

The backpropagation neural network (BPNN) is a neural network training algorithm for financial forecasting, which has powerful problem-solving ability. Multilayer perceptron (MLP) is one of the most prevalent neural networks, which has the capability of complex mapping between inputs and outputs that makes it possible to approximate nonlinear function. Reference [13] employs MLP in trading and hybrid time-varying leverage effects and [14] in forecasting of time series. The two architectures have at least three layers. The first layer is called the input layer (the number of its nodes corresponds to the number of explanatory variables). The last layer is called the output layer (the number of its nodes corresponds to the number of response variables). An intermediary layer of nodes, the hidden layer, separates the input from the output layer. Its number of nodes defines the amount of complexity which the model is capable of fitting. In previous studies, forward networks have frequently been used for financial time series prediction, while, unlike forward networks, recurrent neural network uses feedback connections to model spatial as well as temporal dependencies between input and output series to make the initial states and the past states of the neurons capable of being involved in a series of processing. References [15–17] show the applications in different areas of recurrent neural network. This ability makes them applicable to time series prediction with satisfactory prediction results [18]. As a special recurrent neural network, the Elman recurrent neural network (ERNN) has been used in the present paper for prediction. ERNN is a time-varying predictive control system that was developed with the ability to keep memory of recent events in order to predict future output.

The nonlinear and nonstationary characteristics of the stock market make it difficult and challenging for forecasting stock indices in a reliable manner. Particularly, in the current stock markets, the rapid changes of trading rules and management systems have made it difficult to reflect the markets' development using the early data. However, if only the recent data are selected, a lot of useful information (which the early data hold) will be lost. In this research, a stochastic time effective neural network (STNN) and the corresponding learning algorithm were presented. References [19–22] introduce the corresponding stochastic time effective models and use them to predict financial time series. Particularly, [23] has shown a random data-time effective radial basis function neural network, which is also applied to the prediction of financial price series. The present paper has optimized the ERNN model which is different with the above models; also at first step of the procedures we employ different input variables from [23]. At the last section of this paper, two new error measure methods are first introduced to evaluate the better predicting results of the proposed model than other traditional models. For this improved network model, each of historical data is given a weight depending on the time at which it occurs. The degree of impact of historical data on the market is expressed by a stochastic process, where a drift function and the Brownian motion are introduced in the time strength function in order to make the model have the effect of random movement while maintaining the original trend. In the present work, we combine MLP with ERNN and stochastic time effective function to develop a stock price forecasting model, called ST-ERNN.

In order to display that the ST-ERNN can provide a higher accuracy of the financial time series forecasting, we compare the forecasting performance with the BPNN model, the STNN model, and the ERNN model by employing different global stock indices. Shanghai Stock Exchange (SSE) Composite Index, Taiwan Stock Exchange Capitalization Weighted Stock Index (TWSE), Korean Stock Price Index (KOSPI), and Nikkei 225 Index (Nikkei225) are applied in this work to analyze the forecasting models by comparison.

## 2. Proposed Approach

### 2.1. Elman Recurrent Neural Network (ERNN)

The Elman recurrent neural network, a simple recurrent neural network, was introduced by Elman in 1990 [24]. As is well known, a recurrent network has some advantages, such as having time series and nonlinear prediction capabilities, faster convergence, and more accurate mapping ability. References [25, 26] combine Elman neural network with different areas for their purposes. In this network, the outputs of the hidden layer are allowed to feedback onto themselves through a buffer layer, called the recurrent layer. This feedback allows ERNN to learn, recognize, and generate temporal patterns, as well as spatial patterns. Every hidden neuron is connected to only one recurrent layer neuron through a constant weight of value one. Hence the recurrent layer virtually constitutes a copy of the state of the hidden layer one instant before. The number of recurrent neurons is consequently the same as the number of hidden neurons. To sum up, the ERNN is composed of an input layer, a recurrent layer which provides state information, a hidden layer, and an output layer. Each layer contains one or more neurons which propagate information from one layer to another by computing a nonlinear function of their weighted sum of inputs.

**Figure 1:**
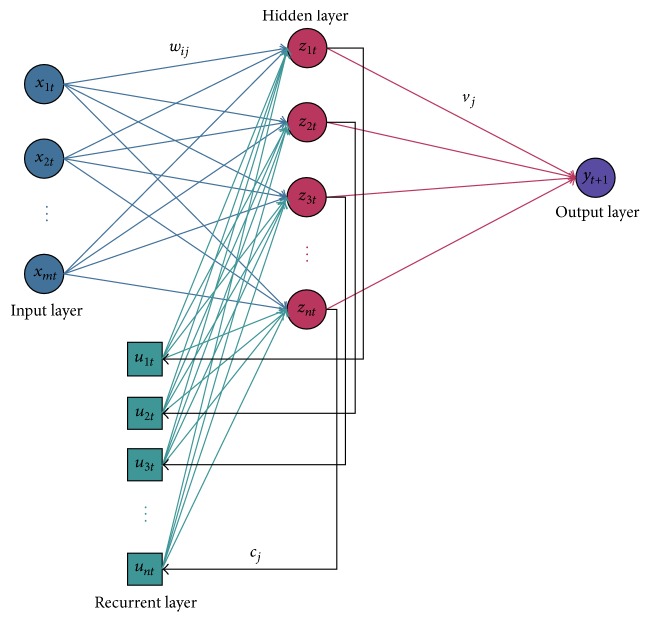
Topology of Elman recurrent neural network.

In Figure 1, a multi-input ERNN model is exhibited, where the number of neurons in inputs layer is *m* and in the hidden layer is *n* and one output unit. Let *x*_*it*_ (*i* = 1,2,…, *m*) denote the set of input vector of neurons at time *t*, *y*_*t*+1_ denotes the output of the network at time *t* + 1, *z*_*jt*_ (*j* = 1,2,…, *n*) denote the output of hidden layer neurons at time *t*, and *u*_*jt*_ (*j* = 1,2,…, *n*) denote the recurrent layer neurons. *w*_*ij*_ is the weight that connects the node *i* in the input layer neurons to the node *j* in the hidden layer. *c*_*j*_, *v*_*j*_ are the weights that connect the node *j* in the hidden layer neurons to the node in the recurrent layer and output, respectively. Hidden layer stage is as follows: the inputs of all neurons in the hidden layer are given by

(1) netjtk=∑i=1nwijxitk−1+∑j=1mcjujtk,ujtk=zjtk−1,  i=1,2,…,n,  j=1,2,…,m.

The outputs of hidden neurons are given by

(2) zjtkfHnetjtk=fH∑i=1nwijxitk+∑j=1mcjujtk,

where the sigmoid function in hidden layer is selected as the activation function: *f*_*H*_(*x*) = 1/(1 + *e* ^−*x*^). The output of the hidden layer is given as follows:

(3) $$\begin{array}{c} y_{t+1}\left(\;k\;\right)=f_{T}\left(\;\overset{m}{\underset{j=1}{\sum }}v_{j}z_{jt}\left(\;k\;\right)\;\right), \end{array}$$

where *f*_*T*_(*x*) is an identity map as the activation function.

### 2.2. Algorithm of ERNN with a Stochastic Time Effective Function (ST-ERNN)

The backpropagation algorithm is a supervised learning algorithm which minimizes the global error *E* by using the gradient descent method [18, 21]. For the ST-ERNN model, we assume that the error of the output is given by *ɛ*_*t*_*n*__ = *d*_*t*_*n*__ − *y*_*t*_*n*__ and the error of the sample *n* is defined as

(4) $$\begin{array}{c} E\left(\;t_{n}\;\right)=\frac{1}{2}\varphi \left(\;t_{n}\;\right){\left(\;d_{t_{n}}-y_{t_{n}}\;\right)}^{2}, \end{array}$$

where *t*_*n*_ is the time of the sample *n* (*n* = 1,…, *N*), *d*_*t*_*n*__ is the actual value, *y*_*t*_*n*__ is the output at time *t*_*n*_, and *φ*(*t*_*n*_) is the stochastic time effective function which endows each historical data with a weight depending on the time at which it occurs. We define *φ*(*t*_*n*_) as follows:

(5) $$\begin{array}{c} \varphi \left(\;t_{n}\;\right)=\frac{1}{\beta }\exp\left\{\;\overset{t_{n}}{\underset{t_{0}}{\int }}\mu \left(\;t\;\right)dt+\overset{t_{n}}{\underset{t_{0}}{\int }}\sigma \left(\;t\;\right)dB\left(t\right)\;\right\}, \end{array}$$

where *β* (>0) is the time strength coefficient, *t*_0_ is the time of the newest data in the data training set, and *t*_*n*_ is an arbitrary time point in the data training set. *μ*(*t*) is the drift function, *σ*(*t*) is the volatility function, and *B*(*t*) is the standard Brownian motion.

Intuitively, the drift function is used to model deterministic trends, the volatility function is often used to model a set of unpredictable events occurring during this motion, and Brownian motion is usually thought as random motion of a particle in liquid (where the future motion of the particle at any given time is not dependent on the past). Brownian motion is a continuous time stochastic process, and it is the limit of or continuous version of random walks. Since Brownian motion's time derivative is everywhere infinite, it is an idealised approximation to actual random physical processes, which always have a finite time scale. We begin with an explicit definition. A Brownian motion is a real-valued, continuous stochastic process {*Y*(*t*), *t* ≥ 0} on a probability space (*Ω*, *ℱ*, *ℙ*), with independent and stationary increments. In detail, we have the following: (a) continuity: the map *s* ↦ *Y*(*s*) is continuous *ℙ* a.s.; (b) independent increments: if *s* ≤ *t*, *Y*_*t*_ − *Y*_*s*_ is independent of *ℱ* = (*Y*_*u*_, *u* ≤ *s*); (c) stationary increments: if *s* ≤ *t*, *Y*_*t*_ − *Y*_*s*_ and *Y*_*t*−*s*_ − *Y*_0_ have the same probability law. From this definition, if {*Y*(*t*), *t* ≥ 0} is a Brownian motion, then *Y*_*t*_ − *Y*_0_ is a normal random variable with mean *rt* and variance *σ* ^2^ *t*, where *r* and *σ* are constant real numbers. A Brownian motion is standard (we denote it by *B*(*t*)) if *B*(0) = 0  *ℙ* a.s., *𝔼*[*B*(*t*)] = 0, and *𝔼*[*B*(*t*)]^2^ = *t*. In the above random data-time effective function, the impact of the historical data on the stock market is regarded as a time variable function; the efficiency of the historical data depends on its time. Then the corresponding global error of all the data at each network repeated training set in the output layer is defined as

(6) E=1N∑n=1NEtn=12N·∑n=1N1βexp⁡∫t0tnμtdt+∫t0tnσtdBt·dtn−ytn2.

The main objective of learning algorithm is to minimize the value of cost function *E* until it reaches the preset minimum value *ξ* by repeated learning. On each repetition, the output is calculated and the global error *E* is obtained. The gradient of the cost function is given by Δ*E* = ∂*E*/∂*W*. For the weight nodes in the input layer, the gradient of the connective weight *w*_*ij*_ is given by

(7) $$\begin{array}{c} \Delta w_{ij}=-\eta \frac{\partial E\left(t_{n}\right)}{\partial w_{ij}}=\eta {ɛ}_{t_{n}}v_{j}\varphi \left(\;t_{n}\;\right)f_{H}^{\prime }\left(\;{\text{net}}_{jt_{n}}\;\right)x_{it_{n}}, \end{array}$$

for the weight nodes in the recurrent layer, the gradient of the connective weight *c*_*j*_ is given by

(8) $$\begin{array}{c} \Delta c_{j}=-\eta \frac{\partial E\left(t_{n}\right)}{\partial c_{j}}=\eta {ɛ}_{t_{n}}v_{j}\varphi \left(\;t_{n}\;\right)f_{H}^{\prime }\left(\;{\text{net}}_{jt_{n}}\;\right)u_{jt_{n}}, \end{array}$$

and for the weight nodes in the hidden layer, the gradient of the connective weight *v*_*j*_ is given by

(9) $$\begin{array}{c} \Delta v_{j}=-\eta \frac{\partial E\left(t_{n}\right)}{\partial v_{j}}=\eta {ɛ}_{t_{n}}\varphi \left(\;t_{n}\;\right)f_{H}\left(\;{\text{net}}_{jt_{n}}\;\right), \end{array}$$

where *η* is the learning rate and *f*_*H*_′(net_*jt*_*n*__) is the derivative of the activation function. So the update rules for the weights *w*_*ij*_, *c*_*j*_, and *v*_*j*_ are given by

(10) wijk+1wijk+Δwijk=wijk+ηɛtnvjφtnfH′netjtnxitn,cjk+1cjk+Δcjk=cjk+ηɛtnvjφtnfH′netjtnujtn,vjk+1vjk+Δvjk=vjk+ηɛtnφtnfHnetjtn.

**Figure 2:**
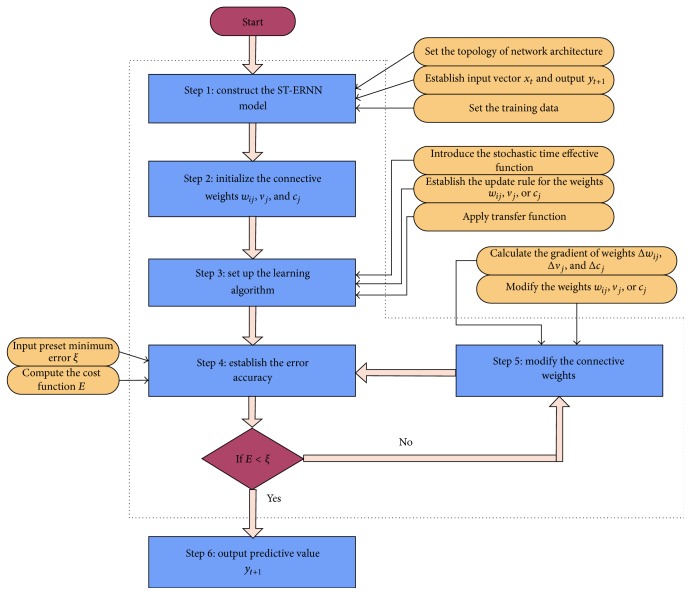
Training algorithm procedures of ST-ERNN.

Note that the training aim of the stochastic time effective neural network is to modify the weights so as to minimize the error between the network's prediction and the actual target. In Figure 2, the training algorithm procedures of the stochastic time effective neural network are displayed, which are as follows.* Step  1.* Perform input data normalization. In ST-ERNN model, we choose four kinds of stock prices as the input values in the input layer: daily opening price, daily highest price, daily lowest price, and daily closing price. The output layer is the closing price of the next trading day. Then determine parameters of the network such as learning rate *η* which is between 0 and 1, the maximum training iterations number *K*, and initial connective weights. Also, the topology of the network architecture is the number of neural nodes in the hidden layer in this paper.* Step  2.* At the beginning of data processing, connective weights *w*_*ij*_, *v*_*j*_, and *c*_*j*_ follow the uniform distribution on (−1,1).* Step  3.* Introduce the stochastic time effective function *φ*(*t*) in the error function *E*. Choose the drift function *μ*(*t*) and the volatility function *σ*(*t*). Give the transfer function from the input layer to the hidden layer and the transfer function from the hidden layer to the output layer.* Step  4.* Establish an error acceptable model and set preset minimum error *ξ*. Based on network training objective *E* = (1/*N*)∑_*n*=1_^*N*^ *E*(*t*_*n*_), if *E* is below preset minimum error, go to Step  6; otherwise go to Step  5.* Step  5.* Modify the connective weights: calculate the gradient of the connective weights *w*_*ij*_, Δ*w*_*ij*_^*k*^, *v*_*j*_, Δ*v*_*j*_^*k*^, *c*_*j*_, and Δ*c*_*j*_^*k*^. Then modify the weights from the layer to the previous layer, *w*_*ij*_^*k*+1^, *v*_*j*_^*k*+1^, or *c*_*j*_^*k*+1^.* Step  6.* Output the predictive value *y*_*t*+1_ = *f*_*T*_(∑_*j*=1_^*m*^ *v*_*j*_ *f*_*H*_(∑_*i*=1_^*n*^ *w*_*ij*_ *x*_*it*_ + ∑_*j*=1_^*m*^ *c*_*j*_ *u*_*jt*_)).

## 3. Forecasting and Statistical Analysis of Stock Price

### 3.1. Selecting and Preprocessing of the Data

To evaluate the performance of the proposed ST-ERNN forecasting model, we select the daily data from Shanghai Stock Exchange (SSE) Composite Index, Taiwan Stock Exchange Capitalization Weighted Stock Index (TWSE), Korean Stock Price Index (KOSPI), and Nikkei 225 Index (Nikkei225) to analyze the forecasting models by comparison. In Table 1 we show that the selected number of each index is 2000. The SSE data cover the time period from 16/03/2006 up to 19/03/2014, the TWSE is from 09/02/2006 up to 19/03/2014, and KOSPI used in this paper is from 20/02/2006 up to 19/03/2014 while Nikkei225 is from 27/01/2006 up to 19/03/2014. Usually, the nontrading time periods are treated as frozen such that we adopt only the time during trading hours. To reduce the impact of noise in the financial market and finally lead to a better prediction, the collected data should be properly adjusted and normalized at the beginning of the modelling. There are different normalization methods that are tested to improve the network training [27, 28], which include “the normalized data in the range of [0,1]” in the following equation, which is also adopted in this work:

(11) $$\begin{array}{c} S{\left(\;t\;\right)}^{\prime }=\frac{S\left(t\right)-\min S\left(t\right)}{\max S\left(t\right)-\min S\left(t\right)}, \end{array}$$

where the minimum and maximum values are obtained on the training set during the training process. In order to obtain the true value after the forecasting, we can revert the output variables as

(12) $$\begin{array}{c} S\left(\;t\;\right)=S{\left(\;t\;\right)}^{\prime }\left(\;\max S\left(\;t\;\right)-\min S\left(\;t\;\right)\;\right)+\min S\left(\;t\;\right). \end{array}$$

### 3.2. Training and Forecasting by ST-ERNN Model

In the ST-ERNN model, after we have done the experiments repeatedly on the different index data, different number of neural nodes in the hidden layer were chose as the optimal; see Table 1. The dataset was divide into two subsets: the training set and the testing set. It is noteworthy that the lengths of the data points we chose for these four time series are the same; the lengths of training data and testing data are also set the same. The training set with 75% of data was used for model building; and the testing set, with the last 25%, was used to test on the out-of-sample set the predictive part of a model. The training set is totally 1500 data, for the SSE is from 16/03/2006 to 22/02/2012 while that for the TWSE is from 09/02/2006 to 08/03/2012, for KOSPI is from 20/02/2006 to 09/03/2012, and for Nikkei is from 27/01/2006 to 09/03/2012. The number of the rest is 500, defined as the testing set. We preset the learning rate and the maximum training cycle by referring to [21, 29, 30]; then we have done the experiment repeatedly on the training set of different index data; different numbers of neural nodes in the hidden layer are chosen as the optimal; see Table 1. The maximum training iterations number *K* is 300, different dataset has different learning rate *η*, and, here, after many times experiments of the training data we choose 0.001, 0.001, 0.05, and 0.01 for SSE, TWSE, KOSPI, and Nikkei225, respectively. And the predefined minimum training threshold *ξ* = 10^−5^. When using the ER-STNN model to predict the daily closing price of stock index, we assume that *μ*(*t*) (the drift function) and *σ*(*t*) (the volatility function) are as follows:

(13) μt=1c−t2,σt=1N−1∑i=1Nx−x¯21/2,

where *c* is the parameter which is equal to the number of samples in the datasets and $\bar{x}$ is the mean of the sample data. Then the corresponding cost function can be written by

(14) E=1N∑n=1NEtn=12N∑n=1N1βexp⁡∫t0tn1c−t2dt+∫t0tn1N−1∑i=1Nx−x¯21/2dBtdtn−ytn2.

**Figure 3:**
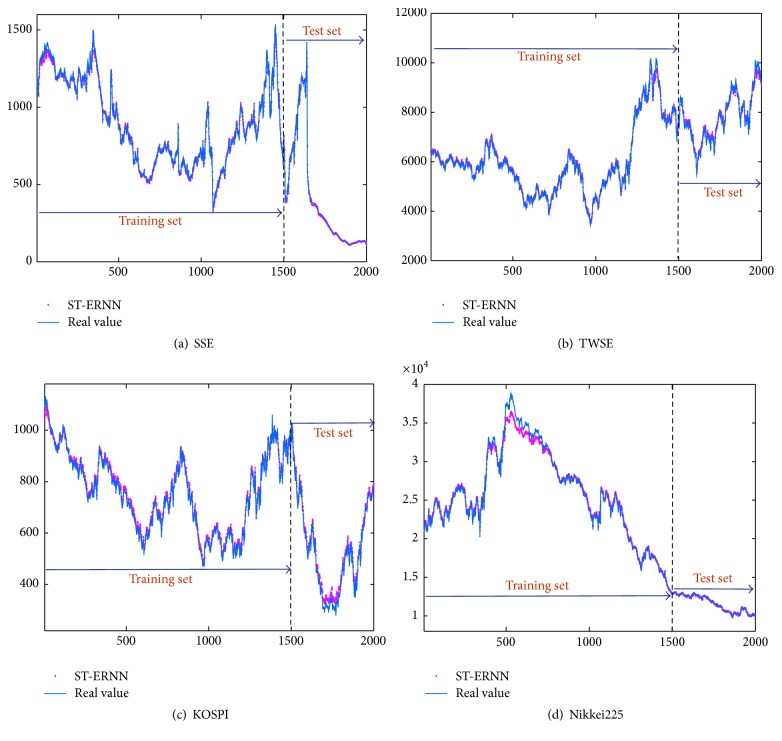
Comparisons of the predictive data and the actual data for the forecasting models.

Figure 3 shows the predicting results of training and testing data for SSE, TWSE, KOSPI, and Nikkei225 with the ST-ERNN model correspondingly. The curves of the actual data and the predictive data are intuitively very approximating. It means that with many times experiments the financial time series have been well trained; the forecasting results are desired by ST-ERNN model.

**Figure 4:**
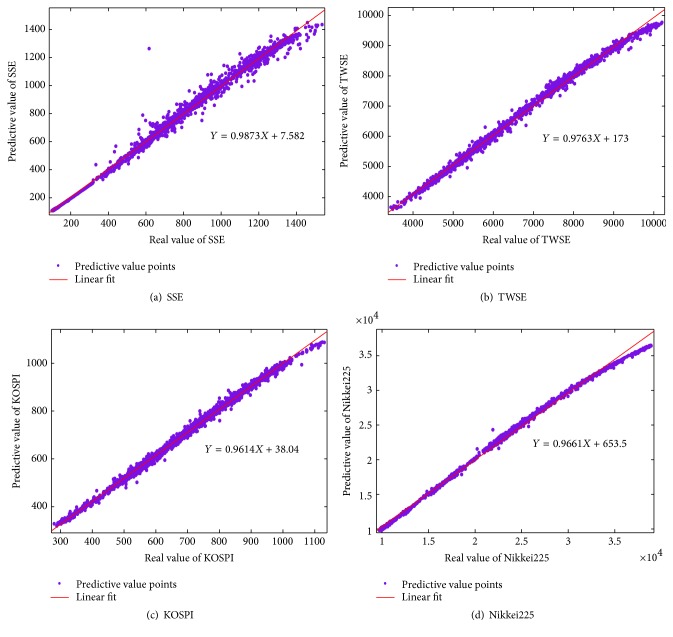
Comparisons and linear regressions of the actual data and the predictive values for SSE, TWSE, KOSPI, and Nikkei225.

The plots of the real and the predictive data for these four price series are, respectively, shown in Figure 4. Through the linear regression analysis, we make a comparison of the predictive value of the ST-ERNN model with the real price data. It is known that the linear regression can be used to fit a predictive model to an observed data set of *Y* and *X*. The linear equations of SSE, TWSE, KOSPI, and Nikkei225 are exhibited, respectively, in Figures 4(a)–4(d). We can observe that all the slopes of the linear equations for them are drawn near to 1, which implies that the predictive values and the real values are not deviating too much.

A valuable numerical measure of association between two variables is the correlation coefficient *R*.  Table 2 shows the values of *a*, *b*, and *R* for the above indices. *R* is given as follows:

(15) $$\begin{array}{c} R=\frac{\sum_{i=1}^{N}\left(d_{t}-{\bar{d}}_{t}\right)\left(y_{t}-{\bar{y}}_{t}\right)}{\sqrt{\sum_{i=1}^{N}{\left(d_{t}-{\bar{d}}_{t}\right)}^{2}\sum_{i=1}^{N}{\left(y_{t}-{\bar{y}}_{t}\right)}^{2}}}, \end{array}$$

where *d*_*t*_ is the actual value, *y*_*t*_ is the predicting value, ${\bar{d}}_{t}$ is the mean of the actual value, ${\bar{y}}_{t}$ is the mean of the predicting value, and *N* is the total number of the data.

### 3.3. Comparisons of Forecasting Results

**Figure 5:**
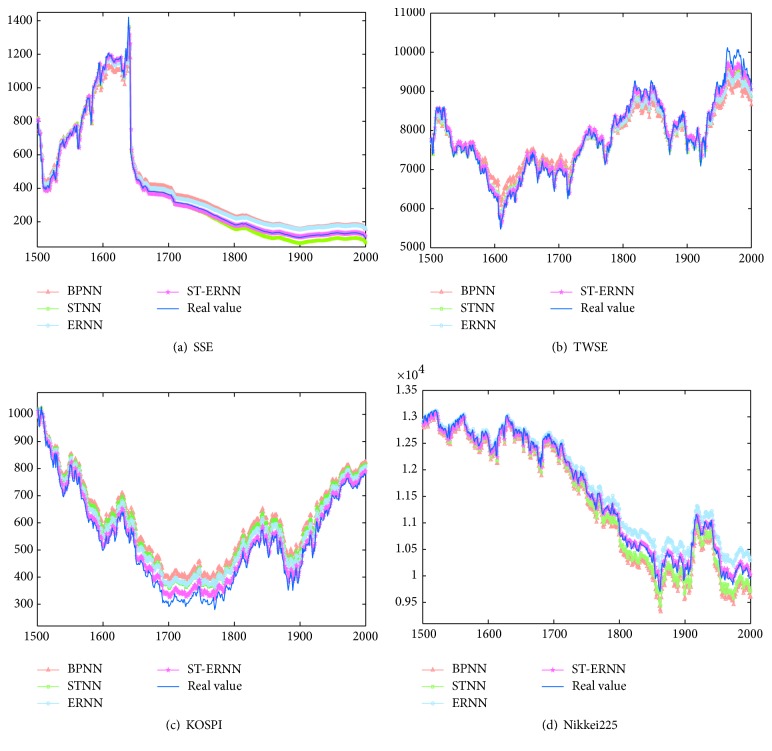
Predictive values on the test set for SSE, TWSE, KOSPI, and Nikkei225.

We compare the proposed and conventional forecasting approaches (BPNN, STNN, and ERNN model) on the four indices mentioned above, where STNN is based on the BPNN and combined with the stochastic effective function [19]. For these four different models, we set the same inputs of the networks, including four kinds of series: daily open price, daily closing price, daily highest price, and daily lowest price. The network output is the closing price of the next trading day. In the stock markets, the practical experience shows us that the above four kinds of data of the last trading day are very important indicators when predicting the closing price of the next trading day. To choose better parameters, we have carried out many experiments on these four different indices. In order to achieve the optimal networks of each forecasting approach, the most appropriate numbers of neural nodes in the hidden layer are different; the learning rates are also varying by training different models; see Table 3. In Table 3, “Hidden” stands for the number of neural nodes in the hidden layer, and “L. r” stands for learning rate. The hidden number is also chosen by referring to [21, 29, 30]. The experiments have been done repeatedly to determine hidden nodes and training cycle in the training process. The details of principles of how to choose the hidden number are as follows: If the number of neural nodes in the input layer is *N*, the number of neural nodes in the hidden layer is set to be nearly 2*N* + 1, and the number of neural nodes in the output layer is 1. Since the ERNN model and the ST-ERNN model have similar topology structures, in Table 3, the number of neural nodes in hidden layer and the learning rate are chosen approximatly in the two models of training process. Also, the BPNN model and the STNN model are similar, so the chosen parameters are basically the same. Figures 5(a)–5(d) show the predicting values of the four indexes on the test set. From these plots, the predicting values of the ST-ERNN model are closer to the actual values than the other models curves. To compare the training and forecasting results more clearly, the performance measures RMSE, MAE, MAPE, and MAPE(100) are uncovered in the next part.

To analyze the forecasting performance of four considered forecasting models deeply, we use the following error evaluation criteria [31–35]: the mean absolute error (MAE), the root mean square error (RMSE), and the correlation coefficient (MAPE); the corresponding definitions are given as follows:

(16) MAE=1N∑t=1Ndt−yt,RMSE=1N∑t=1Ndt−yt2,MAPE=100×1N∑t=1Ndt−ytdt,

where *d*_*t*_ and *y*_*t*_ are the real value and the predicting value at time *t*, respectively. *N* is the total number of the data. Noting that MAE, RMSE, and MAPE are measures of the deviation between the prediction values and the actual values, the prediction performance is better when the values of these evaluation criteria are smaller. However, if the results are not consistent among these criteria, we choose the MAPE as the benchmark since MAPE is relatively more stable than other criteria [16].

**Figure 6:**
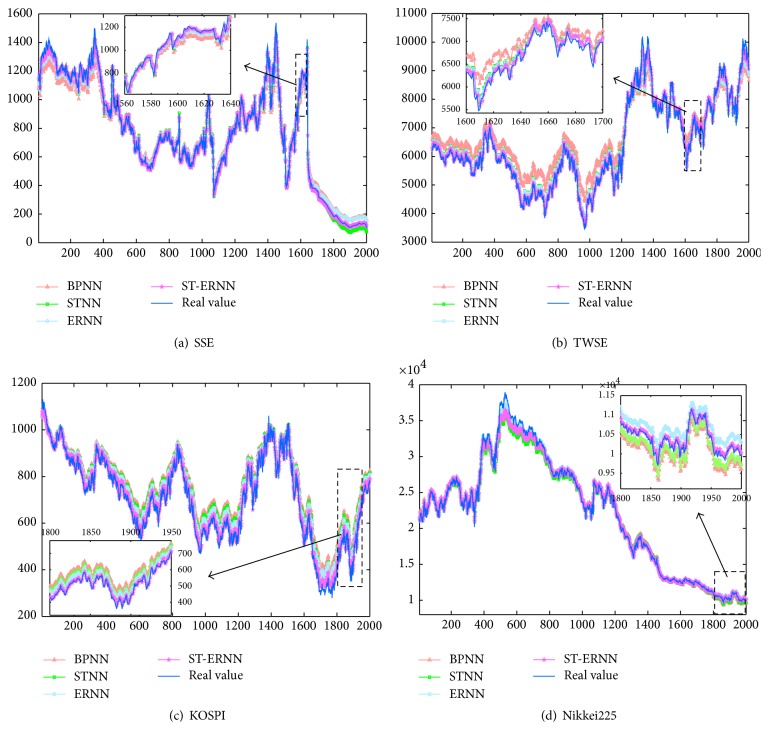
Comparisons of the actual data and the predictive data for SSE, TWSE, KOSPI, and Nikkei225.

Figures 6(a)–6(d) show the forecasting results of SSE, TWSE, KOSPI, and Nikkei225 for four forecasting models. The empirical research shows that the proposed ST-ERNN model has the best performance; the ERNN and the STNN both outperform the common BPNN model. The stock markets showed large fluctuations which are reflected in Figure 6; we can see that the large fluctuation period forecasting is relatively not accurate from these four models. When the stock market is relatively stable, the forecasting result is nearer to the actual value. Compared with the BPNN, the STNN, and the ERNN models, the forecasting results are also presented in Table 4, where the MAPE(100) stands for the latest 100 days of MAPE in the testing data. Table 4 shows that the evaluation criteria by the ST-ERNN model are almost smaller than those by other models. From Table 4 and Figure 6, we can conclude that the proposed ST-ERNN model is better than the other three models. In Table 4, the evaluation criteria by the STNN model and ERNN model are almost smaller than those by BPNN for four considered indices. It illustrates that the effect of financial time series forecasting of STNN model is superior to that of BPNN model, and the dynamic neural network is effective, robust, and precise than original BPNN for these four indices. Besides, most values of MAPE(100) are smaller than those of MAPE in all stock indexes. Therefore, the short-term prediction outperforms the long-term prediction. Overall training and testing results are consistent with the measured data, which demonstrates that the ST-ERNN predictor has higher forecast accuracy.

**Figure 7:**
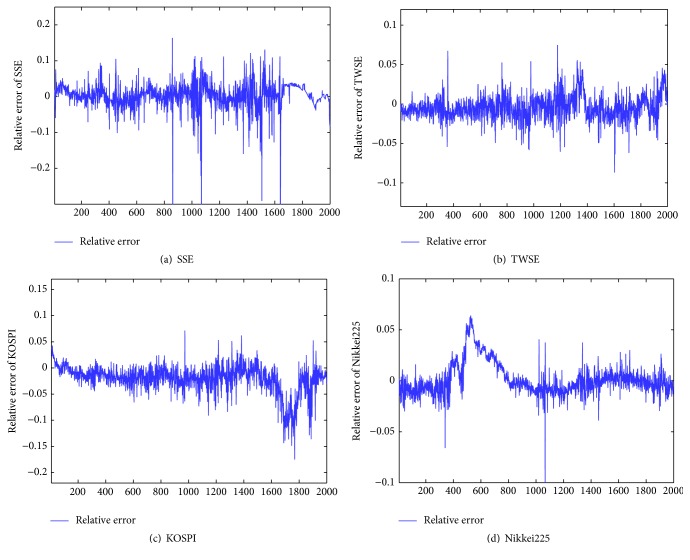
((a), (b), (c), and (d)) Relative errors of forecasting results from the ST-ERNN model.

In Figures 7(a), 7(b), 7(c), and 7(d) we considered the relative errors of the ST-ERNN forecasting results.  Figure 7 depicts that most of the predicting relative errors for these four price series are between −0.1 and 0.1. Moreover, there are some points with large relative errors of forecasting results in four models, especially on the SSE index, which can attribute to the large fluctuation that leads to the large relative errors. The definition of relative error is given as follows:

(17) $$\begin{array}{c} e\left(\;t\;\right)=\frac{d_{t}-y_{t}}{d_{t}}, \end{array}$$

where *d*_*t*_ and *y*_*t*_ denote the actual value and the predicting value, respectively, at time *t*, *t* = 1,2,….

## 4. CID and MCID Analysis

The analysis and forecast of time series have long been a focus of economic research for a more clear understanding of mechanism and characteristics of financial markets [36–42]. In this section, we employ an efficient complexity invariant distance (CID) for time series. Reference [43] shows that complexity invariant distance measure can produce improvements in classification and clustering in the vast majority of cases.

Complexity invariance uses information about complexity differences between two time series as a correction factor for existing distance measures. We begin by introducing Euclidean distance and use this as a starting point to bring in the definition of CID. Suppose we have two time series, *P* and *Q*, of length *n*. Consider

(18) P=p1,p2,…,pi,…,pn,Q=q1,q2,…,qi,…,qn.

The ubiquitous Euclidean distance is

(19) $$\begin{array}{c} \text{ED}\left(\;P,Q\;\right)=\sqrt{\overset{n}{\underset{i=1}{\sum }}{\left(p_{i}-q_{i}\right)}^{2}}. \end{array}$$

The Euclidean distance, ED(*P*, *Q*), between two time series *P* and *Q*, can be made complexity invariant by introducing a correction factor

(20) $$\begin{array}{c} \text{CID}\left(\;P,Q\;\right)=\text{ED}\left(\;P,Q\;\right)\times \text{CF}\left(\;P,Q\;\right), \end{array}$$

where CF is a complexity correction factor defined as

(21) $$\begin{array}{c} \text{CF}\left(\;P,Q\;\right)=\frac{\max\left(\text{CE}\left(P\right),\text{CE}\left(Q\right)\right)}{\min\left(\text{CE}\left(P\right),\text{CE}\left(Q\right)\right)} \end{array}$$

and CE(*T*) is a complexity estimate of a time series *T*, which can be computed as follows:

(22) $$\begin{array}{c} \text{CE}\left(\;T\;\right)=\sqrt{\overset{n-1}{\underset{i=1}{\sum }}{\left(t_{i+1}-t_{i}\right)}^{2}}. \end{array}$$

It is worth noticing that CF accounts for differences in the complexities of the time series being compared. CF forces time series with very different complexities to be further apart. In the case that all time series have the same complexity, CID simply degenerates to Euclidean distance. The prediction performance is better when the CID distance is smaller; that is to say the curve of the predictive data is closer to the actual data. The actual values can be seen as the series *P* and the predicting results as the series *Q*.  Table 5 shows CID distance between the real indices values of SSE, TWSE, KOSPI, and Nikkei225 and the corresponding predictions from each network model. It is clear that the CID distance between the real index values and the prediction by ST-ERNN model is the smallest one; moreover the distances by the STNN model and the ERNN model are smaller than those by the BPNN for all the four considered indices.

In general, the complexity of a real system is not constrained to a sole scale. In this part we consider a developed CID analysis, that is, the multiscale CID (MCID). The MCID analysis takes into account the multiple time scales while measuring the predicting results, and it is applied to the stock prices analysis for the actual data and the predicting data in this work. The MCID analysis should comprise two steps. (i) Considering one-dimensional discrete time series {*x*_1_, *x*_2_,…, *x*_*i*_,…, *x*_*N*_}, we construct consecutive coarse-grained time series {*y* ^(*τ*)^}, corresponding to the scale factor *τ*, according to the following formula:

(23) $$\begin{array}{c} y_{j}^{\left(\tau \right)}=\frac{1}{\tau }\overset{j\tau }{\underset{i=\left(j-1\right)\tau +1}{\sum }}x_{i},\;1\leq j\leq \frac{N}{\tau }. \end{array}$$

**Figure 8:**
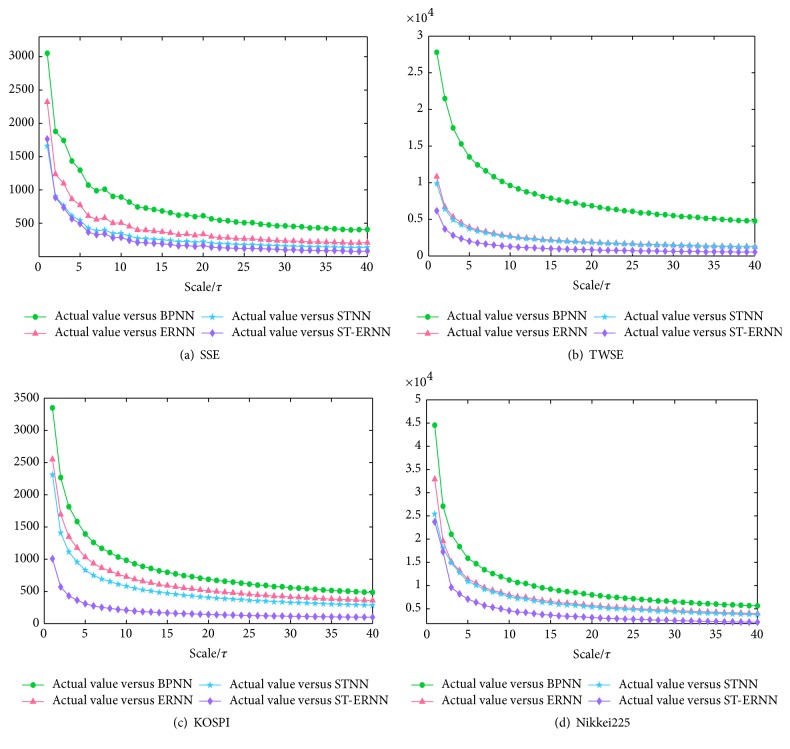
((a), (b), (c), and (d)) MCID values between the forecasting results and the real market prices from BPNN, ERNN, STNN, and ST-ERNN models.

For scale one, the time series {*y* ^(1)^} is simply the original time series. The length of each coarse-grained time series is equal to the original time series divided by the scale factor *τ*. (ii) Calculate the CID for each coarse-grained time series and then plot as a function of the scale factor.  Figure 8 shows the MCID values between the forecasting results and the real market prices from BPNN, ERNN, STNN, and ST-ERNN models. In Figure 8, it is obvious that the MCID from ST-ERNN with the actual value is the smallest one in any scale; that is, the ST-ERNN (with the stochastic time effective function) for forecasting stock prices is effective.

## 5. Conclusion

The aim of this research is to develop a predictive model to forecast the financial time series. In this study, we have developed a predictive model by using an Elman recurrent neural network with the stochastic time effective function to forecast the indices of SSE, TWSE, KOSPI, and Nikkei225. Through the linear regression analysis, it implies that the predictive values and the real values are not deviating too much. Then we take the proposed model compared with BPNN, STNN, and ERNN forecasting models. Empirical examinations of predicting precision for the price time series (by the comparisons of predicting measures as MAE, RMSE, MAPE, and MAPE(100)) show that the proposed neural network model has the advantage of improving the precision of forecasting, and the forecasting of this proposed model much approaches to the real financial market movements. Furthermore, from the curve of the relative error, it can make a conclusion that the large fluctuation leads to the large relative errors. In addition, by calculating CID and MCID distance the conclusion was illustrated more clearly. The study and the proposed model contribute significantly to the time series literature on forecasting.

## Figures and Tables

**Table 1:** Data selection.

Index	Date sets	Total number	Hidden number	Learning rate
SSE	16/03/2006~19/03/2014	2000	9	0.001
TWSE	09/02/2006~19/03/2014	2000	12	0.001
KOSPI	20/02/2006~19/03/2014	2000	10	0.05
Nikkei225	27/01/2006~19/03/2014	2000	10	0.01

**Table 2:** Linear regression parameters of market indices.

Parameter	SSE	TWSE	KOSPI	Nikkei225
*a*	0.9873	0.9763	0.9614	0.9661
*b*	7.582	173	38.04	653.5
*R*	0.992	0.9952	0.9963	0.9971

**Table 3:** Different parameters for different models.

Index data	BPNN		STNN		ERNN		ST-ERNN	
	Hidden	L. r	Hidden	L. r	Hidden	L. r	Hidden	L. r
SSE	8	0.01	8	0.01	10	0.001	9	0.001
TWSE	10	0.01	10	0.01	12	0.001	12	0.001
KOSPI	8	0.02	8	0.02	10	0.03	10	0.05
Nikkei225	10	0.05	10	0.05	10	0.01	10	0.01

**Table 4:** Comparisons of indices' predictions for different forecasting models.

Index errors	BPNN	STNN	ERNN	ST-ERNN
SSE				
MAE	45.3701	24.9687	37.262647	12.7390
RMSE	54.4564	40.5437	49.3907	37.0693
MAPE	20.1994	11.8947	18.2110	4.1353
MAPE(100)	5.0644	3.6868	4.3176	2.6809

TWSE				
MAE	252.7225	140.5971	151.2830	105.6377
RMSE	316.8197	186.8309	205.4236	136.1674
MAPE	3.2017	1.7303	1.8449	1.3468
MAPE(100)	2.2135	1.1494	1.3349	1.2601

KOSPI				
MAE	74.3073	56.3309	47.9296	18.2421
RMSE	77.1528	58.2944	50.8174	21.0479
MAPE	16.6084	12.4461	10.9608	4.2257
MAPE(100)	7.4379	5.9664	4.9176	2.1788

Nikkei225				
MAE	203.8034	138.1857	166.2480	68.5458
RMSE	238.5933	169.7061	207.3395	89.0378
MAPE	1.8556	1.2580	1.5398	0.6010
MAPE(100)	0.7674	0.5191	0.4962	0.4261

**Table 5:** CID distances for four network models.

Index	BPNN	STNN	ERNN	ST-ERNN
SSE	3052.1	1764.7	2320.2	1659.9
TWSE	27805	9876.3	10830	6158.1
KOSPI	3350.4	2312.8	2551.0	1006.0
Nikkei225	44541	23726	32895	25421
